# Nanoparticles systemically biodistribute to regenerating skeletal muscle in DMD

**DOI:** 10.1186/s12951-023-01994-0

**Published:** 2023-08-29

**Authors:** Michael R. Hicks, Xiangsheng Liu, Courtney S. Young, Kholoud Saleh, Ying Ji, Jinhong Jiang, Michael R. Emami, Ekaterina Mokhonova, Melissa J. Spencer, Huan Meng, April D. Pyle

**Affiliations:** 1 Department of Medicine, David Geffen School of Medicine at UCLA, Los Angeles, CA USA; 2 California Nanosystems Institute at UCLA, Los Angeles, CA USA; 3 Department of Microbiology, Immunology and Medical Genetics, David Geffen School of Medicine at UCLA, Los Angeles, CA USA; 4 Eli and Eli and Edythe Broad Center of Regenerative Medicine and Stem Cell Research, Los Angeles, CA USA; 5 Department of Neurology, David Geffen School of Medicine at UCLA, Los Angeles, CA USA; 6 Department of Physiology and Biophysics, University of California Irvine, Irvine, CA USA; 7 Present Address: Zhejiang Cancer Hospital, Hangzhou Institute of Medicine (HIM), Chinese Academy of Sciences, Hangzhou, 310022 Zhejiang China; 8 Present Address: MyoGene Bio, San Diego, CA USA; 9 Present Address: CAS Key Laboratory for Biomedical Effects of Nanomaterials and Nanosafety, National Center for Nanoscience and Technology, Beijing, China

## Abstract

Skeletal muscle disease severity can often progress asymmetrically across muscle groups and heterogeneously within tissues. An example is Duchenne Muscular Dystrophy (DMD) in which lack of dystrophin results in devastating skeletal muscle wasting in some muscles whereas others are spared or undergo hypertrophy. An efficient, non-invasive approach to identify sites of asymmetry and degenerative lesions could enable better patient monitoring and therapeutic targeting of disease. In this study, we utilized a versatile intravenously injectable mesoporous silica nanoparticle (MSNP) based nanocarrier system to explore mechanisms of biodistribution in skeletal muscle of *mdx* mouse models of DMD including wildtype, dystrophic, and severely dystrophic mice. Moreover, MSNPs could be imaged in live mice and whole muscle tissues enabling investigation of how biodistribution is altered by different types of muscle pathology such as inflammation or fibrosis. We found MSNPs were tenfold more likely to aggregate within select *mdx* muscles relative to wild type, such as gastrocnemius and quadriceps. This was accompanied by decreased biodistribution in off-target organs. We found the greatest factor affecting preferential delivery was the regenerative state of the dystrophic skeletal muscle with the highest MSNP abundance coinciding with the regions showing the highest level of embryonic myosin staining and intramuscular macrophage uptake. To demonstrate, muscle regeneration regulated MSNP distribution, we experimentally induced regeneration using barium chloride which resulted in a threefold increase of intravenously injected MSNPs to sites of regeneration 7 days after injury. These discoveries provide the first evidence that nanoparticles have selective biodistribution to skeletal muscle in DMD to areas of active regeneration and that nanoparticles could enable diagnostic and selective drug delivery in DMD skeletal muscle.

**Supplementary Information:**

The online version contains supplementary material available at 10.1186/s12951-023-01994-0.

## Introduction

Skeletal muscle injuries are often characterized by heterogeneity which includes regionalization of inflammatory lesions, sites of regeneration, and spared functional myofibers [1, 2]. Moreover, there is wide variation in the clinical presentation, age of onset, and disease progression for the over 300 muscle disorders described to date, which differentially affect muscles of the head, trunk, or limb [3, 4]. The most prevalent pediatric muscle wasting disease, Duchenne muscular dystrophy (DMD), is a lethal X-linked disorder that affects all muscles due to mutations in the gene encoding dystrophin and is an example of a disorder that does not affect all muscles equally. DMD muscles, such as the diaphragm and quadriceps are more susceptible to disease [5, 6], and diseased areas, as measured by MRI varies throughout the length of dystrophic muscles [7]. Current standards of care would benefit from strategies aimed to more rigorously monitor affected regions of the approximate 640 muscles in the human body and would better inform on therapeutic interventions to specific regenerative or degenerative sites of dystrophic or myopathic muscles [8].

One potential strategy is use of nanoparticles to assist in disease diagnoses as these have been intensively studied in other indications such as cancer [9]. Nanoparticle based therapeutics have been successfully applied in cancer after the first FDA approved liposome nanocarrier emerged in 1995 [10]. However, use of nano-therapeutics beyond cancer remains largely unexplored. This is particularly true for skeletal muscles which are frequently regarded as a tissue type with limited targeting efficiency due to low nanoparticle access [11] and because nanoparticles are frequently uptaken and cleared through the liver and spleen [12]. Under healthy physiological conditions, fenestrations in lungs, abdominal aorta, muscles, and brain are not large enough to allow transvascular escape of MSNPs [13]. The anatomy and structure of skeletal muscle forms tight connections between the inner lining of endothelial cells and the surrounding basal lamina and pericytes, and this regulates muscle capillary integrity [14–16]. However, in pathological conditions, skeletal muscle blood vessel structure, integrity, and permeability are reported to be compromised [17, 18], which prompted us to test whether dystrophic skeletal muscle would be suitable for systemic nanoparticle delivery. Since skeletal muscle composes more than 40% of human body weight, an ideal nanoparticles platform should be suitable for systemic administration such as intravenous injection (IV).

Mesoporous silica nanoparticles (MSNPs) are a multifunctional delivery system that has been shown in vitro and in vivo to be capable of delivering chemotherapeutic agents, small molecules, and proteins to a broad range of diseases including cancer and infectious diseases [19–23]. The multi-functionality of MSNPs provide the possibility of efficient payload encapsulation composed of various chemical structures with the additional power to image the delivery site of interest [22, 24–27]. In spite of the advances using early stage formulations, there has been limited success in outcome efficacy for nano gene delivery for skeletal muscle in vivo [28–30] and there have been no studies to evaluate nano carriers for diagnostic purposes in skeletal muscles. To determine optimal MSNP systematic delivery to skeletal muscle, we coated the silica surface with a uniform and intact lipid bilayer (LB) coating, which sustains long circulatory half-life, improves colloidal stability, and has high biological compatibility [25–27, 31–33]. To determine if the disease state could affect MSNP delivery, we evaluated MSNP biodistribution in mouse models of DMD (*mdx* and the more severe *mdx*-DBA/2) and in immunocompromised DMD models (mdx-NSG and *mdx*-DBA/2 -NSG) that are common models used to assess therapeutics and cell delivery, and we rationalized that immunocompromised mice would improve our understanding of the role of the immune system in MSNP targeting. In brief, we found an increased ability of MSNPs to home to skeletal muscles in dystrophic models and MSNP delivery was preferentially distributed to sites of active muscle degeneration/regeneration. This work suggests MSNPs could serve as a novel diagnostic and delivery tool for modulating muscle damage sites during regeneration and provide a potential new imaging modality across muscle disorders.

## Results

### MSNPs increase biodistribution to dystrophic muscles compared to normal muscles in multiple DMD murine models

We selected MSNPs with a primary diameter of 70 nm as our group has previously optimized this size for systemic penetration into solid tumors [25, 26, 34]. To track biodistribution of MSNPs at the tissue and cellular level in dystrophic muscles, purified MSNPs were surface functionalized with amino groups which enabled conjugation of amine reactive near-infrared (NIR) dye. Further coating MSNPs with uniform lipid bilayers endowed the nanocarriers with enhanced colloidal stability and minimized non-specific adsorption of serum proteins by nanoparticles in blood and prolong their circulation time [33].

**Fig. 1:**
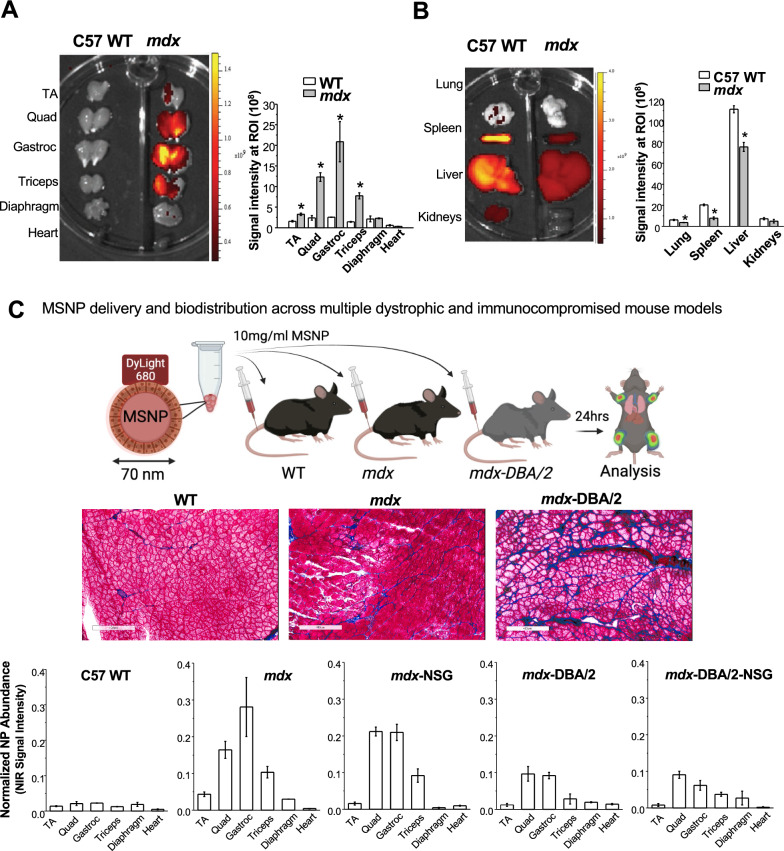
IV injection of MSNPs biodistribute in dystrophic muscles of mdx mice but not normal muscle tissues in wide type mice. **A** and **B** Ex vivo NIR fluorescence imaging of muscles and organs at 24 h after animals received a single IV injection of NIR-labeled lipid coated 70 nm MSNPs (50 mg/kg). NIR fluorescence intensity at ROIs was used to quantify the nanoparticle content in different muscles and organs. Data represent mean ± SEM (n = 3). *P < 0.05, 2-tailed Student’s *t* test. **C-middle** Masson’s Trichome staining of TA and Gastroc in 3-month old male C57BL/6, mdx mice and mdx-D2 mice to demonstrate the level of fibrosis with collagen deposit (blue color) in muscles of the different mouse models. Bars represents 100 μm. **C-bottom** Relative biodistribution of NPs at muscle sites in C57BL/6 WT and different DMD mouse models at 24 h after received IV injection of NIR labeled NPs. The fluorescence intensities of muscles were normalized to the liver for each animal. Data represent mean ± SEM (n = 3)

To evaluate MSNP biodistribution, we performed a comparative analysis on age matched healthy C57BL/6 (WT) versus mdx mice. Mice received a single IV injection of 10 mg/mL NIR dye labeled MSNPs at 50 mg silica dose per kg through the tail vein and were sacrificed 24 h post injection. Skeletal and cardiac muscles were dissected and immediately imaged ex vivo using the IVIS imaging system (Fig. 1A). Contrary to the distribution in wildtype animals, we observed preferential and significantly higher MSNP retention in all limb skeletal muscles of dystrophic mice. This profile was completely changed in wildtype muscles in which low NIR signal was detected. MSNPs showed strong NIR fluorescence intensity in upper and lower limb skeletal muscles including gastrocnemius, quadriceps, and triceps exhibiting 6–tenfold greater MSNP abundance compared to wildtype mice, *p* < *0.05*. Quantitative analysis of signal intensity also showed that MSNP distribution is muscle type dependent. Gastrocnemius (gastroc) and quadriceps produced 3–5 folder higher signal than tibialis anterior (TA) from the same mice. The NIR signals in the diaphragm and heart were weakest suggesting MSNP retention in cardiac and skeletal muscles occur through separate mechanisms.

In addition to muscles, we also harvested the major organs of the reticuloendothelial system (RES; (liver and spleen)), kidneys, and lungs (Fig. 1B). While IV injection of MSNPs led to significant RES organ uptake in normal mice, we found a significant reduction in RES uptake in mdx mice. Most notably, MSNP signal was reduced in the spleen and liver by threefold and 1.5-fold in mdx mice, respectively. This is the first demonstration of a nanoparticle to selectively target dystrophic skeletal muscles as compared to off-target RES organs, presumably due to the competitive MSNP retention within dystrophic mdx muscles (Fig. 1B).

### MSNP bio-distribution is reduced in fibrotic muscle sites but is not affected in immunocompromised mice

The increased MSNP biodistribution in *mdx* skeletal muscle prompted us to ask if the preferential MSNP uptake and retention is dependent upon the disease state of skeletal muscle. We hypothesized that increased disease severity would result in greater MSNP delivery to the muscles. Accordingly, we conducted biodistribution studies in *mdx*-DBA/2 mice, a more fibrotic and severely diseased model which better recapitulates several of the human characteristics of DMD myopathology [35, 36]. Similar to previous, *mdx*-DBA/2 mice were injected with NIR Dye-conjugated MSNPs and after 24 h skeletal muscle and major organs were dissected and imaged with IVIS (Fig. 1C). Surprisingly, however, we found more abundant MSNP retention in mdx models compared to the more severe *mdx*-DBA/2 models.

We next used *mdx* and *mdx*-DBA/2 mice that were crossed to immunocompromised (Nod-Scid-Gamma (NSG)) mice, deficient in T-cell, B-cell, and NK-cells, to test if MSNP biodistribution was regulated by the immune system [37]. We found no significant differences in MSNP biodistribution in *mdx*-NSG compared to mdx mice, and no significant difference between *mdx*-DBA/2-NSG compared *mdx*-DBA/2 mice (Fig. 1C). Rather, differences were primarily observed between the mouse strains and not due to the absence of an adaptive immune system. Abundant MSNP biodistribution to skeletal muscle was confirmed in cross sections of both mouse models using NIR immunofluorescence, as well as laminin and a blank Cy3 channel which accounted for background (Fig. 1C).

### MSNP biodistribution did not correlate with numbers of skeletal muscle capillaries and blood vessels across muscle groups nor dystrophic mouse models

**Fig. 2:**
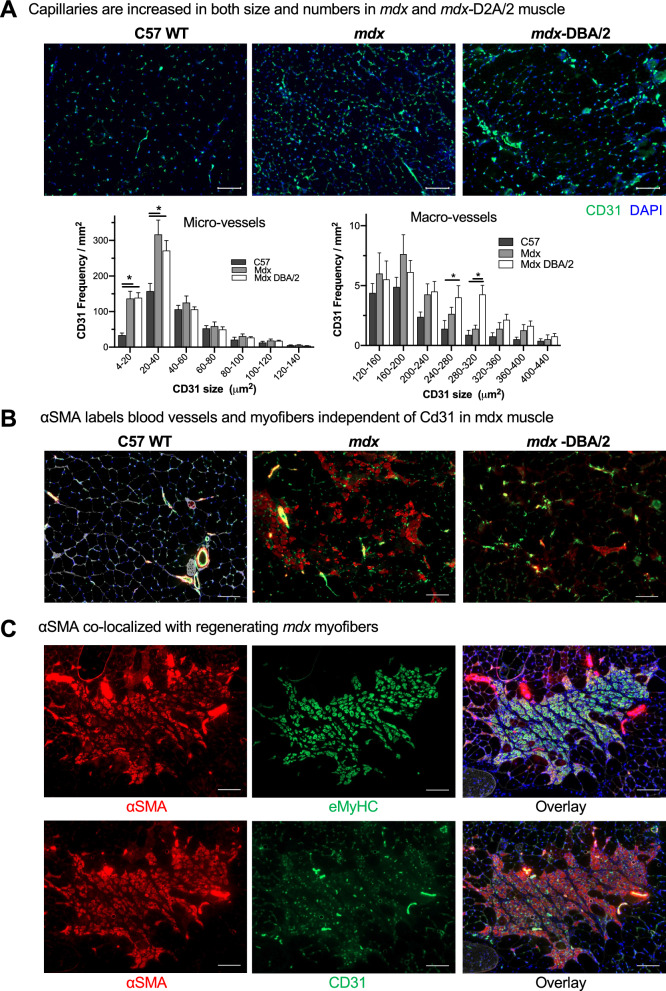
Dystrophic muscles differ in their degenerative/regenerative states which correlates to MSNP biodistribution. **A.** Representative microscopy images of CD31 immunofluorescence (IF) staining of tibialis anterior (TA) and gastrocnemius (Gastroc) in 3-months male C57BL/6 mice, *mdx* and *mdx*-DBA/2 mice. Color code: green, Cd31; blue, nuclear. Scale bar represents 50 μm. Histogram analysis of Cd31 + blood vessel density and size shows quantification from more than 8 randomly selected regions. Data represent mean ± SEM. *P < 0.05, 1-way ANOVA followed by Tukey’s test. **B.** αSMA labels blood vessels and myofibers independent of Cd31 in *mdx* muscle. Bars represents 100 μm. **C.** αSMA co-localizes with regenerating *mdx* myofibers. Scale Bar represents 50 μm

As nanoparticles historically do not biodistribute efficiently to skeletal muscle [38, 39], we set out to identify the mechanism for MSNP accumulation in skeletal muscles and determine if we could further improve muscle homing. We first quantified blood vessel numbers (marked by Cd31) and cross-sectional area from quadriceps of C57BL/6 WT, *mdx*, and *mdx*-DBA/2 mice. We found an increased number of blood vessels in both the *mdx* and *mdx*-DBA/2 mice compared to age-matched WT muscle, *p* < *0.05*. Specifically, CD31 positive micro-vessels (between 4 and 40 μm^2^) increased between dystrophic and wildtype mice (Fig. 2A). However, the differences in micro-vessel density between *mdx* and *mdx*-DBA/2 mice were not statistically different, and thus alone could not account for biodistribution changes between these mouse models. In fact, *mdx*-DBA/2 mice had a greater number of large vessels (between 200 and 300 μm^2^), which did not correlate with increased MSNP biodistribution to the less dystrophic *mdx* mice.

In parallel, we investigated why specific skeletal muscles such as quadriceps and gastrocnemius had increased MSNP numbers compared to tibialis anterior. We found no significant differences or trends in endothelial cell density, pericyte coverage, or trichrome staining between gastrocnemius and tibialis anterior in *mdx* or *mdx*-DBA/2 mice that accounted for the 6–tenfold MSNP increase in gastrocnemius biodistribution (Additional file 1: Fig. S1). We next hypothesized that muscle-specific functional use and blood perfusion [40] may differentially regulate MSNP biodistribution, and thus performed a more detailed vasculature analysis using endothelial (CD31) and smooth muscle (α-SMA) colocalization to distinguish large blood vessels from capillaries. In wildtype mice, CD31 and α-SMA consistently colocalized demarcating large blood vessels as expected (Fig. 2B). Interestingly, in the *mdx* mice we found a striking increase in α-SMA non-colocalized CD31. In *mdx* mice, the α-SMA + staining was specific to centrally nucleated myofibers, which is a hallmark of muscle regeneration myofibers (Fig. 2B, C). However, in *mdx*-DBA/2 muscle α-SMA + myofibers were greatly reduced (Fig. 2B). Previous reports have shown α-SMA is transiently expressed by regenerating myofibers [41], thus we stained muscle serial sections for the regenerative marker embryonic myosin (eMyHC). Indeed in *mdx*, we found dozens of α-SMA + myofibers co-localized with eMyHC + (Fig. 2C). Thus, while we originally hypothesized changes in blood vessel density would increase MSNP biodistribution, we instead identified that changes in muscle regeneration directly corresponded with increased MSNP biodistribution across multiple mouse models.

### MSNPs biodistribute to sites of regenerating skeletal muscle

**Fig. 3:**
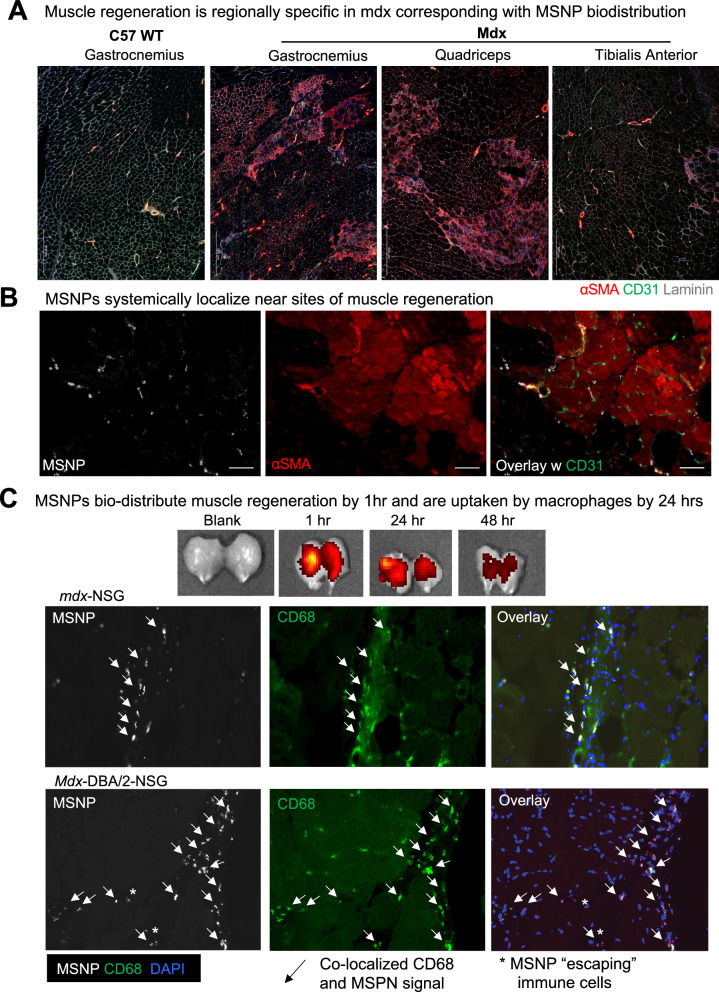
MSNPs biodistribute to regenerating myofibers in dystrophic muscle. **A–B**. Staining of muscle regeneration. Tiled images of gastrocnemius, quadriceps, and tibialis anterior muscles shows varying levels of regeneration across and corresponds with MSNP biodistribution. Scale bars represents 500 μm. **B–C**. Time course of MSNP biodistribution to skeletal muscle. MSNPs packaged with NIR labeled protein bio-distribute to skeletal muscle by 1 h and are cleared by 48 h. IVIS of skeletal muscle tissue at 1, 24, and 48 h post MSNP systemic delivery (50 mg/kg). Staining shows MSNPs (white), regenerating myofibers (a-SMA, red), capillaries (Cd31, green), and nuclei (DAPI, blue). Scale bars represent 20 μm. Color code: Red, vascular smooth-muscle cells stained with anti-alpha smooth muscle actin (α-SMA) antibody, nuclear stained with DAPI; white, NIR MSNPs. Co-staining of Cd68 + macrophages (green) with MSNPs (white) shows greater than 90% co-localization at 24 h post systemic injection

We next evaluated sites of muscle regeneration across the muscle groups: gastrocnemius, quadriceps, tibialis anterior in *mdx* and wildtype mice. Just as NIR-labeled MSNP had enhanced biodistributed to gastrocnemius and quadriceps, we found  a higher frequency of α-SMA + myofibers within these muscles and fewer α-SMA + myofibers in *mdx* TA muscles. We have included zoomed out tiled images that show this substantial increase in α-SMA + myofibers (Fig. 3A-B). Unlike *mdx*, C57 wildtype gastrocnemius showed no α-SMA + myofibers which also correlated with the little-to-no uptake of MSNPs.

To confirm increased MSNP biodistribution at sites containing regenerating myofibers, we again delivered NIR-labeled MSNPs to *mdx* mice and after 24 h evaluated muscle cross sections for co-localization of nanoparticles with CD31 or α-SMA + or eMYHC + Laminin + myofibers. As anticipated, we found increased MSNPs at sites of α-SMA + myofibers (Fig. 3A). This is important given the cellular composition of dystrophic skeletal muscle is heterogeneous containing region of regeneration, necrosis, and relatively normal myofibers, and we found increased NIR-labeled MSNPs aggregated near sites of regenerating myofibers. We therefore concluded that MSNPs predominately bio-distribute within tissues to specific sites of regenerating myofibers which could be due to the MSNPs entering the muscle after injury, and regeneration following muscle injury.

### MSNPs are detected in regenerating muscle by 1 h and rapidly cleared by macrophages

To determine dynamics of MSNPs entering and clearance from the muscle, we next performed a time course analysis of MSNP biodistribution. For these studies, a protein standard cytochrome-c was labeled with NIR dye and packaged in MSNPs for analysis. By IVIS imaging, we demonstrated MSNPs can be robustly detected within skeletal muscle by 1 h and the signal is significantly decreased within 48 h of systemic injection (Fig. 3C). Skeletal muscles were dissected and cross sections imaged at 1, 4, 24, and 48 h, which found MSNPs within the muscle capillaries at 1 h and existing the vasculature into the muscle stroma by 4 h. By 24 h, greater than 90% of MSNPs were uptaken by CD68 + macrophages in all mouse models including immunocompromised *mdx*-NSG and *mdx*-DBA/2-NSG mice, suggesting that macrophages are playing a role in clearance or transport of MSNPs to regenerative sites in the muscle (Fig. 3C and S3). These results indicate that systemic MSNP injection would enable rapid imaging of damaged muscle and would be transiently retained for short periods of 1–2 days.

### Experimentally induced injury validates MSNPs bio-distribute to regenerating skeletal muscle

We reasoned that if MSNPs biodistributed to sites of muscle regeneration, then we should be able to induce muscle injury and identify MSNPs accumulated at sites of regeneration. Barium chloride (BaCl_2_) is a commonly used agent which robustly induces skeletal muscle regeneration following local intramuscular injection [42]. Immediately following injection, BaCl_2_ blocks calcium channels of skeletal myofibers resulting in myofiber death, local inflammation, and blood vessel fragmentation causing temporary ischemia [43]. The newest regenerating myofibers generated from satellite cell differentiation occur approximately 7 days after injury [44]. To validate whether we could induce a regenerative response with corresponding blood vessel recovery, we performed BaCl_2_ injections and analysis in skeletal muscle of uninjured, 1 day, and 7 days post injury (dpi). At 7 dpi, we identified high numbers of α-SMA + eMyHC + myofibers juxtaposed to dilated endothelial cells marked by Cd31 + α-SMA-, as well as an influx of Cd68 + macrophages juxtaposed to eMyHC + myofibers (Additional file 4: Fig. S4A-B). To show functional vascular perfusion at this time point, we performed the Miles assay using Evan’s Blue dye (EBD) which allows researchers to study vascular hyperpermeability through the proxy measurement of vascular leakage [45], and showed an increased EBD update 1 h after intravenous injection relative to uninjured controls (Additional file 4: Fig. S4C).

**Fig. 4:**
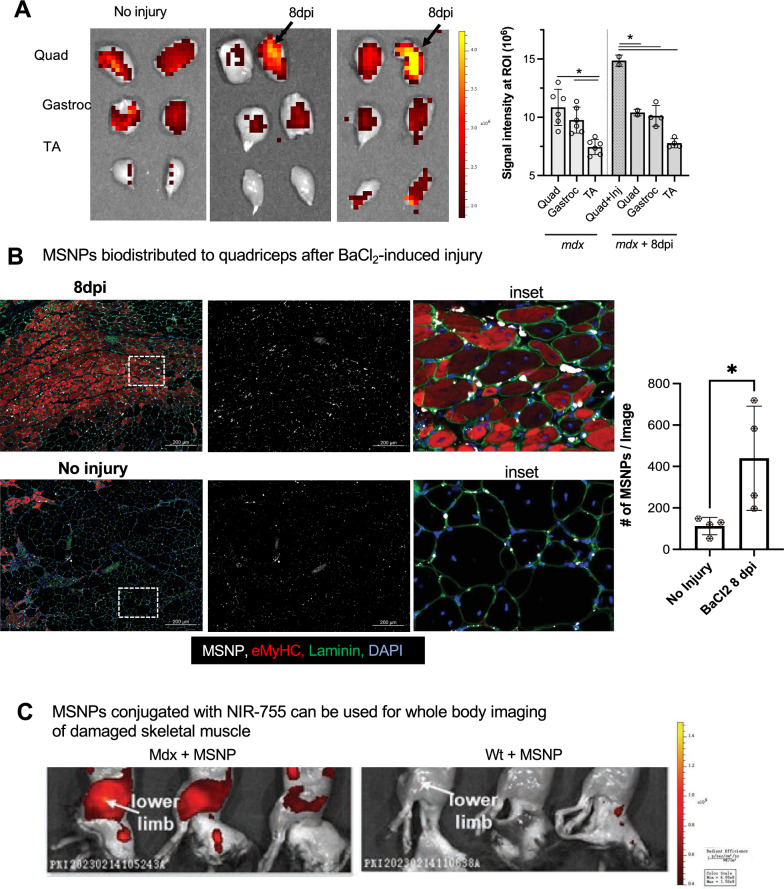
MSNPs biodistributed to quadriceps after experimental-induced injury. **A.** MSNPs were injected 7 days post injury to right *mdx* quadriceps. Ex vivo NIR fluorescence imaging of limb muscles at 24 h after animals received IV injection of NIR-labeled lipid-coated 70 nm MSNPs (50 mg/kg). **B.** Tissue sections and graph show increase accumulation of MSNPs (white) near eMyHC (red) positive myofibers. Graph quantifies, the size and fluorescent intensity of MSNPs in BaCl_2_-injured and non-injured *mdx* quadriceps (N = 3). **C.** Whole body imaging of animals at 24 h after IV injection of DyLight755 labeled MSNPs

After validating muscle regeneration and endothelial cell permeability at this time point, we injected a single quadriceps site with BaCl_2_ and after 7 dpi, delivered NIR-labeled MSNPs to demonstrate diagnostic detection and bio-distribution at regenerative sites. Consistent with our previous data, non-injured mdx mice had robust signal in quadriceps and gastrocnemius, and NIR signal was equally distributed between left and right legs of uninjured *mdx* mice (Fig. 4B). However, in BaCl_2_ treated quadriceps, the MSNPs showed a significant increase in signal relative to other non-injured muscle tissues (*p* < *0.05*, Fig. 4B-C). NIR signaling data was further validated by immunofluorescent staining which showed large numbers of MSNPs interspersed between endothelial cells and laminin basal membrane of eMyHC + myofibers (Fig. 4B). To evaluate whole-body distribution of MSNPs in wild type and dystrophic mice we synthesized NIR Dylight 755 labeled MSNPs which have lower background interference for living animals imaging. We performed the whole-body imaging of the sacrificed animal with hair removal at 24 h post-injection, which demonstrated the nanoparticles selectively accumulated at the muscles in both upper and lower limbs in mdx mice but not in wildtype C57 mice (Fig. 4C).

## Discussion

In this communication, we developed a versatile nanoparticle platform that biodistributes to dystrophic skeletal muscle and the muscle interstitium. Dystrophic skeletal muscle goes through repeated cycles of degeneration and regeneration and is highly inflammatory [46] where damaged or leaky vasculature could provide a route into the muscle for MSNPs, and nanoparticles themselves have been shown to induce vascular leakiness [47]. One key discovery in this work is the contrasting MSNP retention in dystrophic muscles versus normal muscle, accompanied by up to 1–2 log fold reduced nanoparticle content in liver and spleen. Combined use of IVIS imaging and NIR dye-conjugated MSNPs enabled high photon penetration in vivo and this suggests MSNPs could be modified to work in clinical settings such as with MRI to serve as a non-invasive tool for whole-body tracking of damaged skeletal muscle or for delivery of labelled drugs to skeletal muscle, in a similar manner to nanoparticle-mediated identification of tumors in cancer patients [48].

DMD pathophysiology and other muscular dystrophies non-uniformly affects specific muscle groups [4, 6] which may reflect muscle involvement and vulnerability during gait and other activities [49]. Not only do we show that MSNPs preferentially home to dystrophic muscle, but we also show that MSNPs home to muscle groups that are undergoing regeneration. It has been shown that after a muscle injury, capillaries regenerate before, or synchronously with, skeletal muscle and that the initial regenerated capillaries are disorganized and dilated [50]. We likewise show dilated microvasculature adjacent to regenerating myofibers that present a potential route for MSNP transcytosis into muscle. In addition, macrophages have critical roles in muscle regeneration and are found in large numbers at regenerative sites. Macrophages and other innate myeloid cells may also regulate microvascular permeability for nanoparticle delivery [51]. Future studies will need to determine whether macrophages are bringing MSNPs to sites of muscle regeneration or whether MSNP transcytosis preferentially occurs through newly formed microvessels which are then phagocytosed by macrophages. As macrophages are highly interactive with MSNPs this could warrant further development of nano-mediated myeloid cell modulation in skeletal muscle [52, 53]. Recent work has shown that Interleukin(IL)-4 or IL-10 could be readily packaged within MSNPs to regulate anti-inflammatory responses which to improve muscle quality leading to increased contractile force [54].

Interestingly, we did not find MSNPs going to heart which does not regenerate, and found little to no MSNPs within the mdx diaphragm which exhibits a pattern of degeneration, fibrosis and severe functional deficit comparable to that of mdx limb muscles [55]. Similarly, we also found that delivery in the more severely degenerated mdx-DBA/2 mice was compromised compared to standard mdx mice suggesting that fibrosis or lack of regeneration may be a limiting barrier to MSNP delivery. Combination therapies that target the fibrotic nature of skeletal muscle may be needed to improve drug delivery. TGFβ is a well-known regulator of fibrosis and we have shown that inhibition of TGFβ with small molecules can be safely released in skeletal muscle in vitro (Additional file 5: Figure S5) which suggests that MSNPs offer an exciting route for preventing extensive fibrosis. Thus MSNPs could be used in combination to enable targeted therapies to improve site specific regeneration [7]. However, further analysis of the implications for drug delivery using MSNPs are required as it has been shown that depending on the MSNP characteristics such as coating and tissue type, can affect delivery efficiency or vascular toxicity [56, 57].

We demonstrated MSNPs are capable of biodistribution in immunocompromised *mdx*-NSG and *mdx*-DBA/2-NSG mice, and thus MNSPs may be useful for augmenting stem-cell based research and therapies in DMD by providing supportive survival or migratory signal release during engraftment [37, 58–61]. MSNPs are particularly relevant for DMD as there are many therapies that could benefit from combination therapies such as exon skipping where proteins or small molecules can be used to enhance efficiency [62]. Delivery of CRISPR/Cas9 editing machinery using MSNPs remains a challenge for skeletal muscle in vivo*;* however, other nanoparticle based systems such as lipid nanoparticles (LNPs) have been shown to be able to deliver LNPs via limb perfusion to target multiple muscle groups, with low immunogenicity and efficient intramuscular delivery of CRISPR-Cas9 [63]. Systemic delivery and release of gene editing cargo remains a significant challenge for LNPs or MSNPs and currently AAV mediated delivery is the most efficient. It is possible that use of innovative muscle homing peptides could improve delivery to skeletal muscle [64, 65] but will still require optimization for efficient release of cargo into skeletal muscle.

In this work we showed for the first time, the use of a nanoparticle made for diagnostic use at sites of muscle regeneration. MSNPs as a diagnostic could complement multi-slice MRI or CT scans to identify regeneration sites for patients with neuromuscular disease. As porous MSNPs can be functionalized to carry proteins, future work could be tailored for delivery of protein payloads to dystrophic muscle sites. MSNPs accumulation at the site of damage with therapeutic payload could further improve regeneration or target macrophage inflammation. In summary this work suggests that MSNPs may provide a unique avenue for imaging individual diseased muscles and have great potential for identifying and modulating disease progression in DMD or other muscular dystrophies.

## Materials and methods

### Materials

All chemicals were directly used without further purification.

### Animals

All animal work was conducted under protocols approved by the UCLA Animal Research Committee (ARC) in the Office of Animal Research Oversight (OARO). C57BL/10 J (C57, #000665) C57BL/10ScSn-Dmd^mdx^/J (Mdx, #001801), and D2.B10-Dmd^mdx^/J (Mdx-DBA/2, #013141) were purchased from Jackson laboratory. To generate immunocompromised strains, Mdx and mdx-DBA/2 mice were crossed to NOD.Cg-Prkdc^scid^ Il2rg^tm1Wjl^/SzJ (NSG, 005557) mice. Mice containing the severe combined immune deficiency (scid) and a complete null allele of the IL2 receptor common gamma chain (IL2rgnull) were then back crossed to Mdx or Mdx-DBA/2 for at least 5 generations to create congenic strains. All the animals were housed in the Biomedical Sciences Research Building’s Humanized Mouse Core at UCLA, an immunocompromised core facility. All mice experiments were conducted between 2 and 4 months of age, and all experiments were performed on age and sex matched controls. For barium chloride studies, 1.2% BaCl_2_ was diluted in Hanks Buffered Saline Solution (HBSS) and 50 μL injected into quadriceps of anesthetized mice.

### MSNP Synthesis

To study systemic biodistribution of MSNPs to skeletal muscle, near-infrared (NIR) dye labeled monodisperse spherical MSNPs with a primary size of ~ 70 nm with a lipids surface coating were synthesized as described in our previous reports [26, 33]. Briefly, MSNPs were synthesized by a sol–gel chemistry using a seed-growth procedure. 5 mL of CTAC (25 wt% in water) was added to 15 mL of DI H_2_O, followed by stirring at 500 rpm for 10 min at 85 °C. This was followed by the addition of 0.8 mL of 10% (w/v) TEA-ol for 10 min at 85 °C. Then, 1 mL of the silica precursor, TEOS, was added and stirred at 350 rpm at 85 °C for 1 h, leading to the formation particles with a primary size of ∼70 nm. The particles were washed by in acidic ethanol (HCl/ethanol, 4:100 v/v) through a repeated centrifugation and resuspension process to remove the surfactant CTAC as before. Purified MSNPs were labeled by the NIR dye, which were first surface functionalized with NH_2_ groups using APTES and then were conjugated with the NHS ester of NIR fluorescent dye, DyLight 680 NHS ester (0.1 wt% to MSNPs). Last, the NIR labeled MSNPs were coated with a lipid bilayer (LB). Briefly, a mixture of lipids (32 mg DSPC, 10.8 mg, cholesterol (Chol) and 5.4 mg DSPE-PEG_2000_, yielding a DSPC/Chol/DSPE-PEG_2000_ molar ratio of 3:2:0.15) was dissolved in 100 µL pure ethanol at ~ 65 °C. One mL of a preheated solution, containing a 20 mg/mL NIR labeled MSNP suspension into was added into the lipid solution and mixed by pipetting. The mixture was treated by 52 W probe sonication using a 10 s/5 s on/off cycle for 10 min. The particle suspension was purified by centrifugation and washing with PBS. MSNP morphology is characterized using a transmission electron microscope (JEOL 1200-EX) and the coated lipid bilayer on MSNPs was visualized by cryoEM (TF20 FEI Tecnai-G2). The fluorescence spectra of labeled particles were measured by a Microplate Reader (M5e, Molecular Device, USA). Particle concentration, hydrodynamic size, and zeta potential were measured by a ZETAPALS instrument (Brookhaven Instruments Corporation).

For protein standard loading, MSNPs with a pore size of ~ 3 nm were synthesized by applying a heterogeneous oil − water biphase stratification reaction system according to the literature with modification [66, 67]. NIR dye labeled cytochrome-c (10 mg/mL in 10 mM PBS at pH 5) were mixed with bare MSNPs (10 mg/mL in 10 mM PBS at pH 5) in 1:1 ratio, and the mixture was shaken overnight at room temperature. The protein loaded MSNPs were further coated with lipid bilayer similar as shown above. These formulations were used in Figs. 3C and Additional file 3: Fig. S3.

To validate nanoparticle formulations from multiple sources, we purchased custom made MSNPs from NanoComposix, which followed a similar formation by modifying 50 mg of mesoporous silica nanoparticles (75 nm diameter confirmed by TEM) with Dylight™ 680 and deliver the final material in ethanol at a concentration of 10 mg/mL of nanoparticles. These were used in Fig. 4.

### IVIS evaluation of biodistribution of MSNPs across DMD mouse models

All animals received an identical dose of NIR-labeled particles (50 mg/kg MSNPs) by intravenous (IV) injection. After 24 h, animals were sacrificed and followed by ex vivo imaging of the excised different muscles and major organs. All tissues were kept on wet ice chilled glass Petri dishes until dissections were completed, at which time they were transferred to fluorescent blocking paper and placed into a pre-warmed In Vivo Imaging System chamber (IVIS Lumina II, Perkin Elmber) and imaged at the UCLA Preclinical Imaging Technology Center. MSNPs signal intensity was quantified by IVIS Lumina Living Image software version 4.7.3 using radiance efficiency ((Photons/Sec/cm^2^/sr) /μW/cm^2^) to analyze regions of interest. Both left and right muscles limb were used for analysis. In Figure C, for the ease of comparison across animal models, fluorescence intensities of muscles were normalized to the liver for each animal (Fig. 1C).

For biodistribution studies the following mouse numbers were used: Fig. 1 and Additional file 1: Fig. S1: C57BL/6 and *mdx* mice (n = 3 ea.), mdx-DBA/2 (n = 4), and mdx-NSG and mdx-DBA/2-NSG (n = 5 ea.). Time course analysis at 1, 4, 24, and 48 mice used n = 2–6 ea. in Fig. 3 and Additional file 3: Fig. S3. Evan’s blue dye and BaCl_2_ optimizations were performed on no injury, 1 day post injury, and 8 days post injury and used n = 2 *mdx* mice in Additional file 4: Figure S4. These were followed by n = 6 no injury and n = 5 BaCl2 injured *mdx* mice combined with MSNPs in Fig. 4.

Comparative analysis of differences between groups was performed using the 2-tailed Student’s *t*-test (Excel software, Microsoft) for two-group comparison. A One-way ANOVA followed by a Tukey’s test (Origin software, OriginLab and/or GraphPad PRISM) was performed for multiple group comparisons. A One-way ANOVA followed by a Dunnet’s test was performed to compare all muscle groups to injured quadriceps. Data were expressed as mean ± SD or SEM, as stated in the figure legends. A statistically significant difference was considered at *p* < 0.05.

### Immunofluorescent characterization of DMD mouse model tissues and cells

Immediately following IVIS imaging, gastrocnemius, quadriceps, triceps, and tibialis anterior muscles were cryoembedded in tissue plus optimal cutting temperature (O.C.T.) compound and flash frozen in isopentane chilled by liquid nitrogen (LN2) and then stored in isopentane filled scintillation vials at – 80 °C. Muscle tissues were sectioned at − 20 °C using a Leica Cryostat at 10 μm. Immunofluorescent staining included TrueBlack^®^ Lipofuscin Autofluorescence Quencher (Biotium) and blocking buffer (0.2% gelatin, 3% BSA, 10% goat serum, 0.1% tween-20 in PBS) to improve signal to noise ratio in identifying MSNPs by epi-fluorescent (Zeiss Observer-1) and confocal (Zeiss LSM-780 and Leica SP8) microscopy. To analyze deep in the muscle, all tissues were cryosectioned to a depth of 2 mm, and then twelve transverse 10 μm sections were collected for analysis.

Tissues were then stained with various antibodies (Table 1). Four 10X images (0.6 mm^2^) were randomly captured from the upper and lower sectioned regions using laminin as a biomarker. Images co-stained for CD31 were then imported to Imaris Bitplane software (version 9.3.0) which was used to calculate the frequency and size distribution of all CD31-positive staining using Imaris Cell Module. Fluorescence intensity threshold were automatically calculated by Imaris and visually confirmed. All CD31 features greater than 10 μm^3^, where the z-voxel size equals one, were included in analyses. Point spread function was used for segmentation of adjacent objects. For fine tuning, identified CD31 features were exported to Imaris Surface Module, and all images were individually scrutinized for non-specific and auto-fluorescent staining, which allowed for correction of over or under merged identified objects. CD31 data were then exported to GraphPad PRISM. All features 10-120μm^2^ were plotted in a histogram using frequency distribution function for each individual mouse with a bin size set to 10 μm^2^. Statistical differences were calculated using One-way ANOVA with posthoc Tukey for C57BL/6, *mdx*, and *mdx*-DBA/2 mice at each bin size, *p* < *0.05*. For larger blood vessels 120–400 μm^2^ bins were set to 20 μm^2^ and statistical differences similarly calculated.

**Table 1:** Directory of chemicals, reagents and antibodies and vendor information

Product	Vendor	Catalog No
Tetraethylorthosicate (TEOS)	Sigma-Aldrich	86578
Triethanolamine (TEA-ol)	Sigma-Aldrich	90279
Cetyltrimethylammonium chloride solution (CTAC)	Sigma-Aldrich	292737
3-aminopropyl)triethoxysilane (APTES)	Sigma-Aldrich	440140
Gold (III) chloride hydrate (HAuCl_4_·3H_2_O)	Sigma-Aldrich	520918
Trisodium citrate dihydrate	Sigma-Aldrich	S4641
1,2-Distearoyl-sn-glycero-3-phosphocholine (DSPC)	Avanti Polar Lipids	850365
1, 2-distearoyl-sn-glycero-3-phosphoethanolamine-N-[methoxy (polyethylene glycol)-2000] (ammonium salt) (DSPE-PEG_2000_)	Avanti Polar Lipids	880120
Cholesterol (Chol)	Avanti Polar Lipids	700000
Anti-Cd31 antibody	BD Pharmingen	553708
Anti-Cd68 antibody	Bio-Rad	MCA1957
Anti-Laminin antibody	Sigma-Aldrich	L9393
Anti-alpha smooth muscle actin (a-SMA) antibody	Sigma-Aldrich	A5228
Embryonic myosin heavy chain	DSHB	F1.652
Alexa Fluor^®^ 488 conjugated goat anti-rat IgG (H + L) secondary antibody	Thermo Fisher Scientific	A11006
Alexa Fluor^®^ 594 conjugated goat anti-rat IgG (H + L) secondary antibody	Thermo Fisher Scientific	A11007
Alexa Fluor^®^ 488 conjugated goat anti-rabbit IgG (H + L) secondary antibody	Thermo Fisher Scientific	A11008
Alexa Fluor^®^ 594 conjugated goat anti-rabbit IgG (H + L) secondary antibody	Thermo Fisher Scientific	A11012
DyLight 680 NHS ester	Thermo Fisher Scientific	46418

To quantify α-SMA staining, 3 × 2 10X tiled regions (3.2 mm^2^ region) were taken from top and bottom region of gastrocnemius, quadriceps, and tibialis anterior from all mice. To show that α-SMA was in regions of regenerating muscle fibers and this corresponded to the increased MSNP signal found by in situ IVIS imaging, serial sections from the original MSNP size library experiments were stained for α-SMA, embryonic myosin heavy chain and NIR-MSNPs. Co-localization of embryonic myosin heavy and α-SMA were then evaluated using Imaris masking function. Blood vessel endothelial cells and pericytes identified by immunofluorescence staining using anti-CD31 and anti-NG2 antibodies. The fibrosis of muscle tissue was characterized by Masson’s Trichome staining to look at collagen deposit which was performed at UCLA Translational Pathology Core Laboratory (TPCL). Human pluripotent stem cell (hPSC) differentiation, fusion and myosin staining (MF20, DSHB) was performed as previously described [37].

### Supplementary Information

**Additional file 1: Figure S1**. IV injection of MSNPs biodistribute to severely dystrophic mdx-DBA/2 and immunocompromised mdx-NSG mice. **A.** Ex vivo NIR fluorescence imaging of muscles at 24 h after animals received IV injection of NIR-labeled lipids coated 70 nm MSNPs (50 mg/kg). **B.** Immunofluorescent staining of gastrocnemius in mdx-NSG and mdx-DBA/2 mice demonstrate abundant MSNP aggregation in skeletal muscle tissue. Secondary antibody (2y) shows background autofluorescence in skeletal muscle does not co-localize with MSNPs. Scale bars represent 100 μm.

**Additional file 2: Figure S2. **Endothelial cell and pericyte coverage in mdx muscle groups does not correlate with MSNP Biodistribution. **A**. Representative immunofluorescence images of Cd31+ endothelial cells and NG2+ pericytes staining of gastrocnemius, quadriceps, tibialis anterior of *mdx* mice. Graph show total Cd31+ subset by co-localization of Cd31 and NG2 (PC-EC) and single positive Cd31 (EC only) across muscles from C57BL/6 mice, mdx and mdx-D2 mice. Total area was calculated using Imaris and co-localization determined using Imaris masking function. Quantifications were from 6 randomly selected regions, N=3 mice per model. Statistics were performed on total endothelial cell coverage (PC-EC plus EC only), *P < 0.05, 1-way ANOVA multiple comparisons Tukey’s test.

**Additional file 3: Figure S3. **Time course of MSNP biodistribution to skeletal muscle. Images show skeletal muscle tissue at 1, 4, 24, and 48 hrs post MSNP systemic delivery (50 mg/kg). Tissue staining reveals that MSNPs exit the microvasculature at ~4 hrs and are within the interstitial space of skeletal muscle by 24 hrs. Staining shows MSNPs (white), myofibers (laminin, red), capillaries (Cd31, green), and nuclei (DAPI, blue). Scale bars represent 20 μm.

**Additional file 4: Figure S4**. Evaluation of the regenerative microenvironment following BaCl_2_. A. Regenerating mdx myofibers (αSMA, red) are juxtaposed to enlarged, dilated Cd31+ capillaries (green) compared to non-regenerating muscle regions. **B**. Regenerating myofibers contain an influx of macrophages. eMyHC (green), Cd68 (red). Scale bars represent 20 μm. **C**. Images show increased Evan’s Blue Dye at the whole tissue level (blue) and within muscle cross sections (red) at 8 dpi compared to non-injured.

**Additional file 5: Figure S5**. MSNPs do not cause significant toxicity to skeletal muscle *in vitro*. Myogenic cells derived from human pluripotent stem cells (H9 line) were induced to differentiate to myotubes and treated with MSNPs or MSNPs loaded with the TGF-beta inhibitor (SB431542) every two days for 6 days. Myotubes were stained with myosin (MF20) and Image-J used to quantify percent coverage of myosin within the well (N=4). Graph shows that compared to no treatment, that MSNP alone have no effect on myotube differentiation, and that MSNPs containing SB are able to increase myogenic differentiation. * = P<0.05.

## Data Availability

All data and materials are available upon request and with appropriate approvals.
