# Supplementary figures and images for: Nanoparticles systemically biodistribute to regenerating skeletal muscle in DMD
Source: J Nanobiotechnology. 2023 Aug 29;21:303. doi: 10.1186/s12951-023-01994-0 (PMC10463982; doi:10.1186/s12951-023-01994-0)

Figure S1

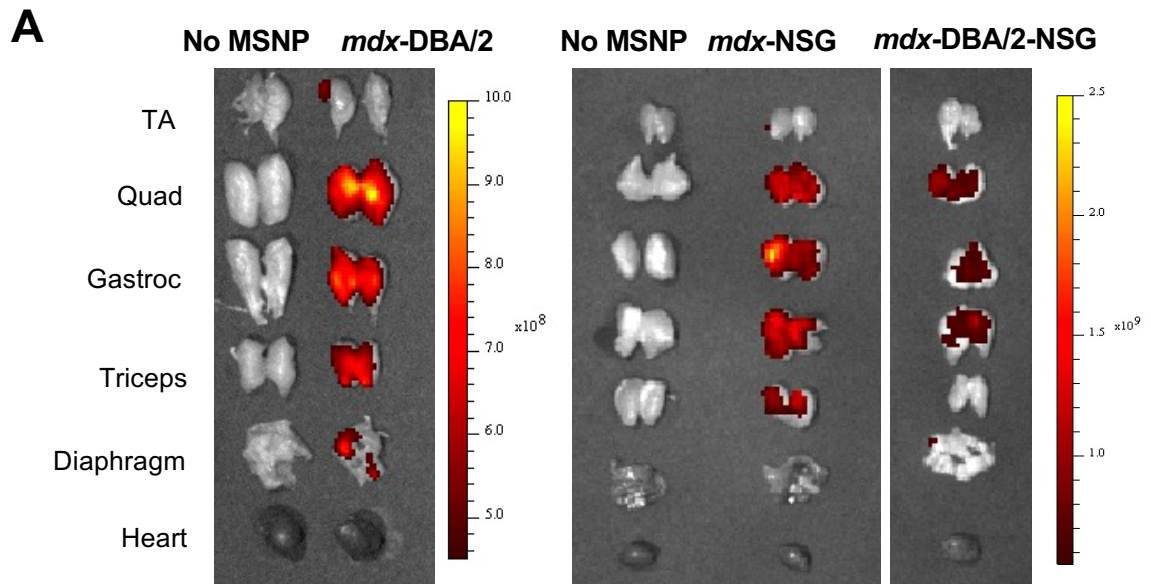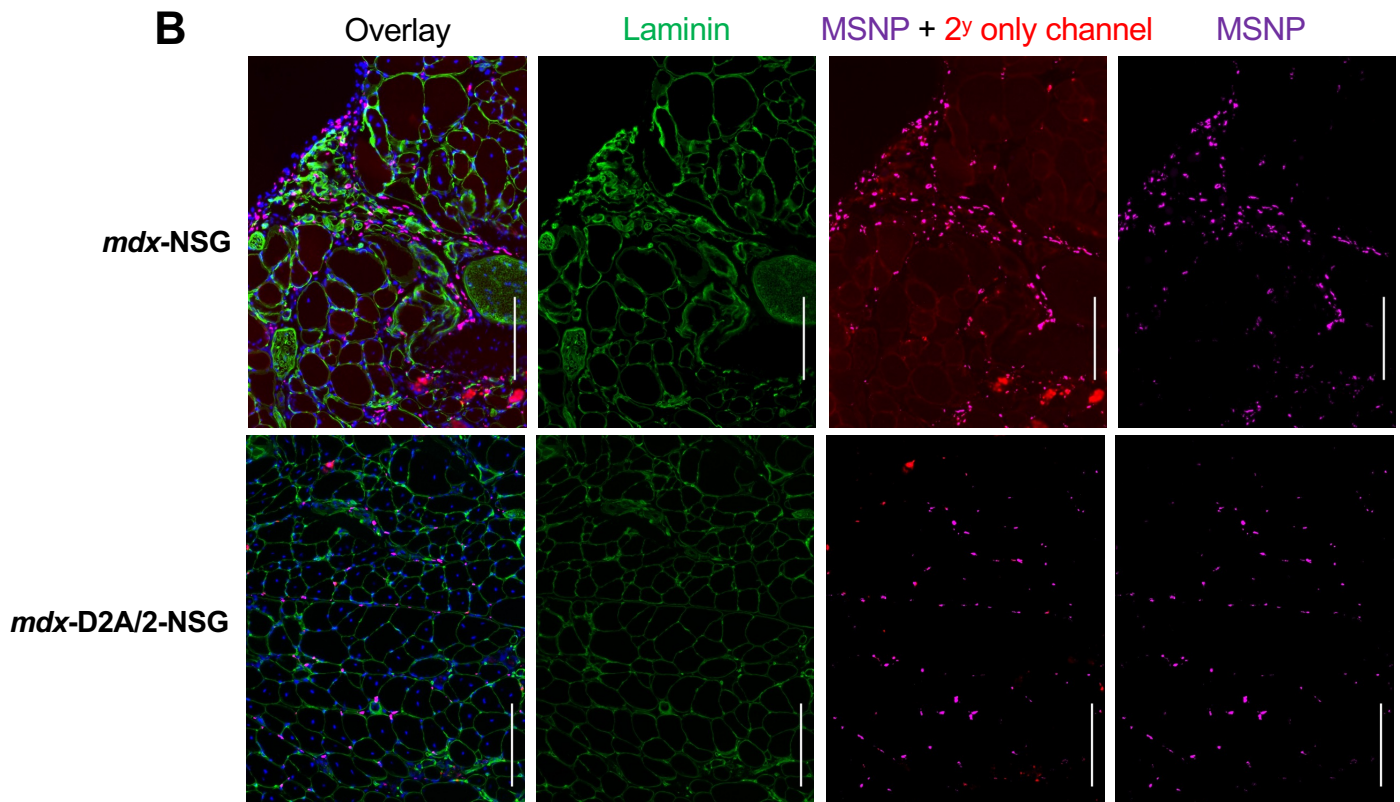

Supplement: Supplementary file 1 — Additional file 1: Figure S1. IV injection of MSNPs biodistribute to severely dystrophic mdx-DBA/2 and immunocompromised mdx-NSG mice. A. Ex vivo NIR fluorescence imaging of muscles at 24 h after animals received IV injection of NIR-labeled lipids coated 70 nm MSNPs (50 mg/kg). B. Immunofluorescent staining of gastrocnemius in mdx-NSG and mdx-DBA/2 mice demonstrate abundant MSNP aggregation in skeletal muscle tissue. Secondary antibody (2y) shows background autofluorescence in skeletal muscle does not co-localize with MSNPs. Scale bars represent 100 μm. [file 12951_2023_1994_MOESM1_ESM.pdf]

# Figure S2

A

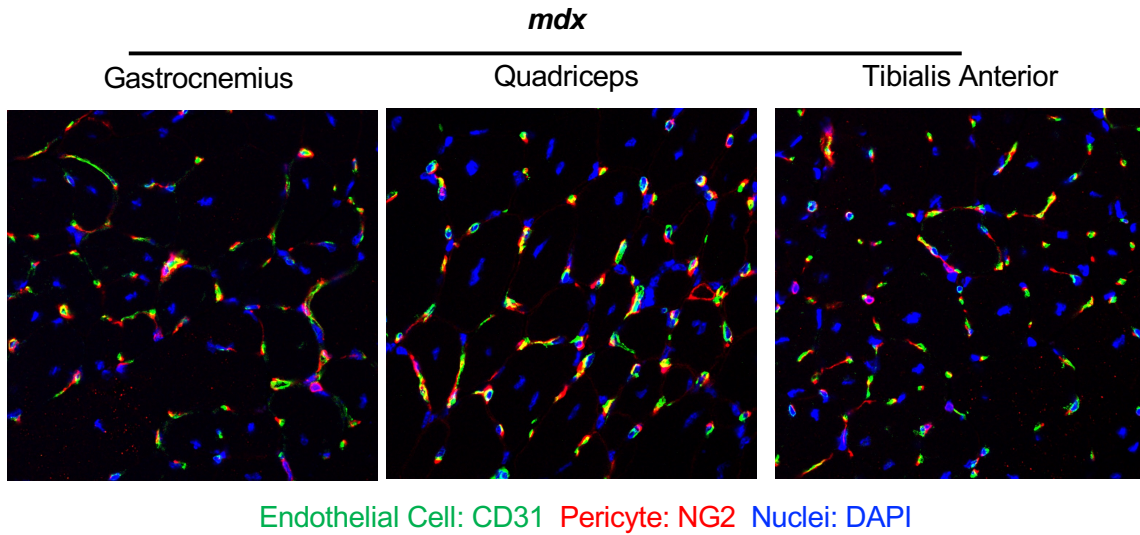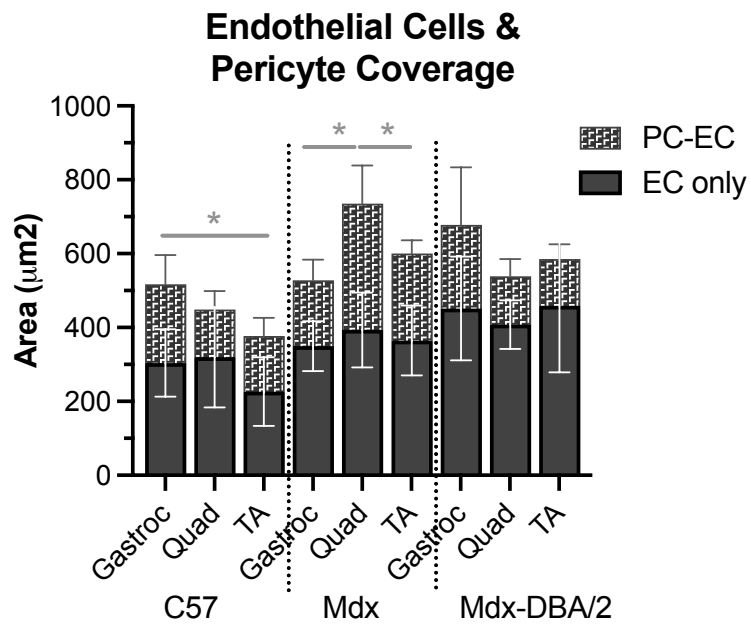

Supplement: Supplementary file 2 — Additional file 2: Figure S2. Endothelial cell and pericyte coverage in mdx muscle groups does not correlate with MSNP Biodistribution. A. Representative immunofluorescence images of Cd31+ endothelial cells and NG2+ pericytes staining of gastrocnemius, quadriceps, tibialis anterior of mdx mice. Graph show total Cd31+ subset by co-localization of Cd31 and NG2 (PC-EC) and single positive Cd31 (EC only) across muscles from C57BL/6 mice, mdx and mdx-D2 mice. Total area was calculated using Imaris and co-localization determined using Imaris masking function. Quantifications were from 6 randomly selected regions, N=3 mice per model. Statistics were performed on total endothelial cell coverage (PC-EC plus EC only), *P < 0.05, 1-way ANOVA multiple comparisons Tukey’s test. [file 12951_2023_1994_MOESM2_ESM.pdf]

**Figure S3**

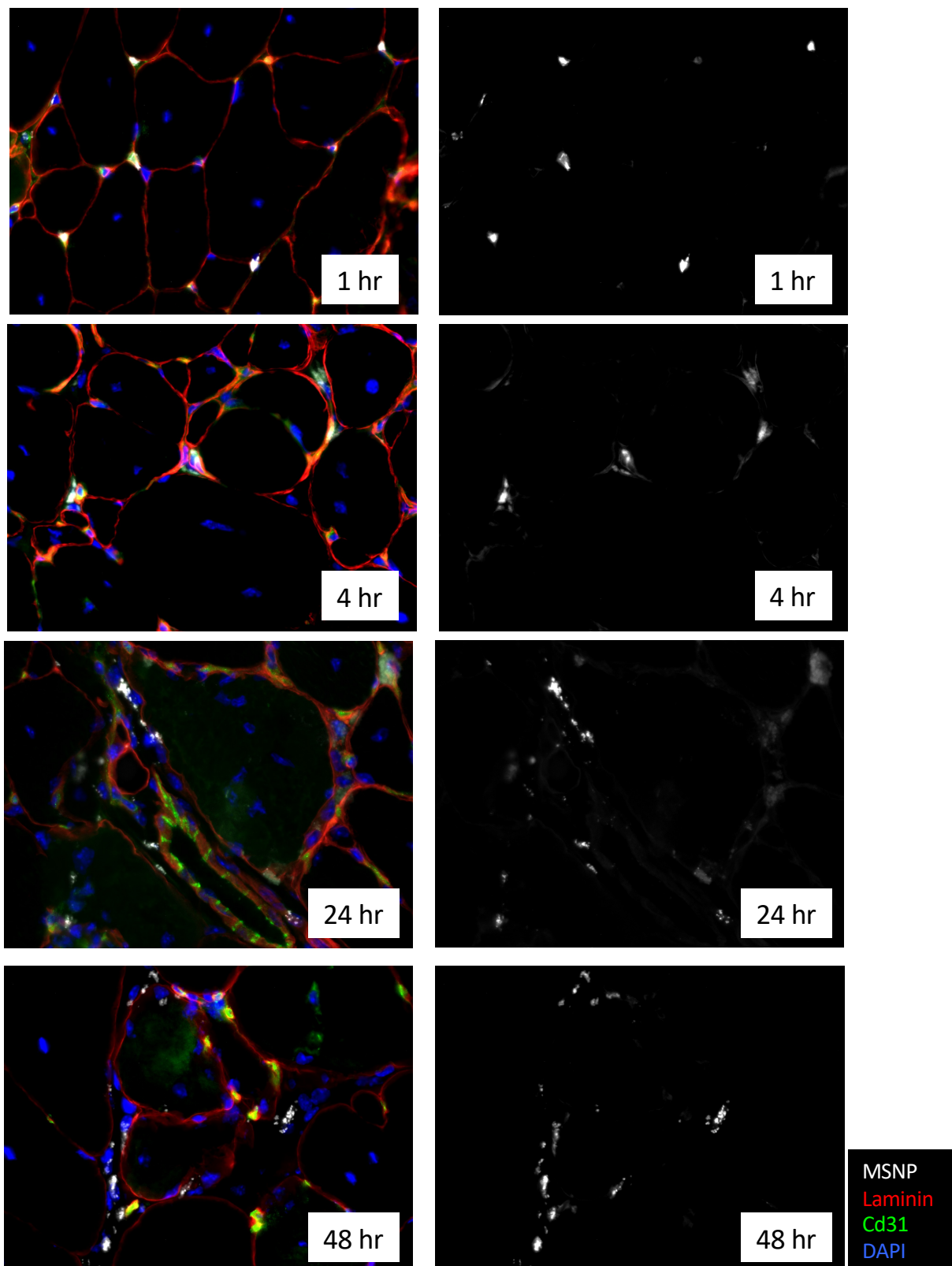

Supplement: Supplementary file 3 — Additional file 3: Figure S3. Time course of MSNP biodistribution to skeletal muscle. Images show skeletal muscle tissue at 1, 4, 24, and 48 hrs post MSNP systemic delivery (50 mg/kg). Tissue staining reveals that MSNPs exit the microvasculature at ~4 hrs and are within the interstitial space of skeletal muscle by 24 hrs. Staining shows MSNPs (white), myofibers (laminin, red), capillaries (Cd31, green), and nuclei (DAPI, blue). Scale bars represent 20 μm. [file 12951_2023_1994_MOESM3_ESM.pdf]

## Slide 1
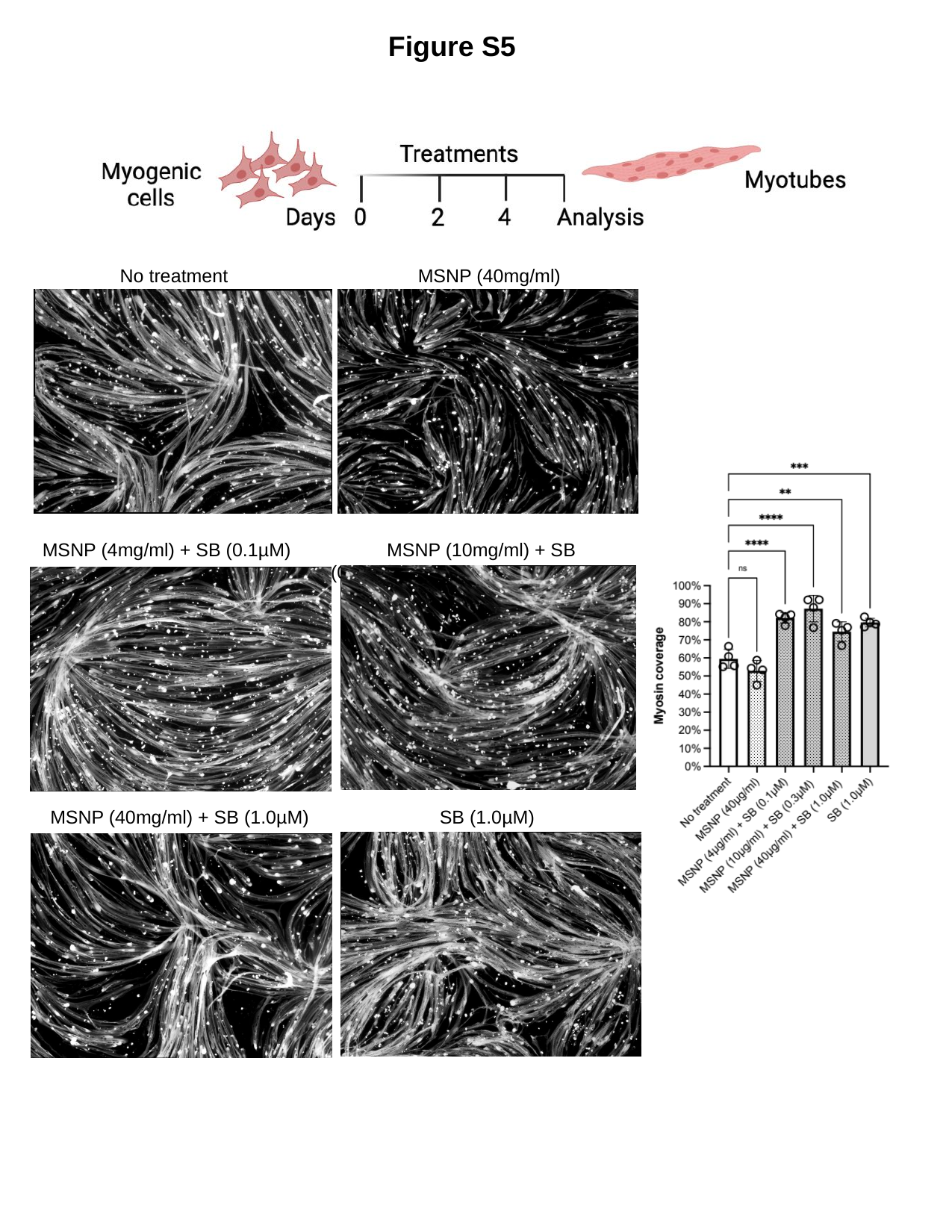

Figure S5
MSNP (40mg/ml)
No treatment
MSNP (4mg/ml) + SB (0.1µM)
MSNP (10mg/ml) + SB (0.3µM)
MSNP (40mg/ml) + SB (1.0µM)
SB (1.0µM)

Supplement: Supplementary file 5 — Additional file 5: Figure S5. MSNPs do not cause significant toxicity to skeletal muscle in vitro. Myogenic cells derived from human pluripotent stem cells (H9 line) were induced to differentiate to myotubes and treated with MSNPs or MSNPs loaded with the TGF-beta inhibitor (SB431542) every two days for 6 days. Myotubes were stained with myosin (MF20) and Image-J used to quantify percent coverage of myosin within the well (N=4). Graph shows that compared to no treatment, that MSNP alone have no effect on myotube differentiation, and that MSNPs containing SB are able to increase myogenic differentiation. * = P<0.05. [file 12951_2023_1994_MOESM5_ESM.pptx]
