# Supplementary material for: Nanoparticles systemically biodistribute to regenerating skeletal muscle in DMD
Source: J Nanobiotechnology. 2023 Aug 29;21:303. doi: 10.1186/s12951-023-01994-0 (PMC10463982; doi:10.1186/s12951-023-01994-0)

Figure S4

A Regenerating skeletal muscle contain dilated Cd31+ capillaries

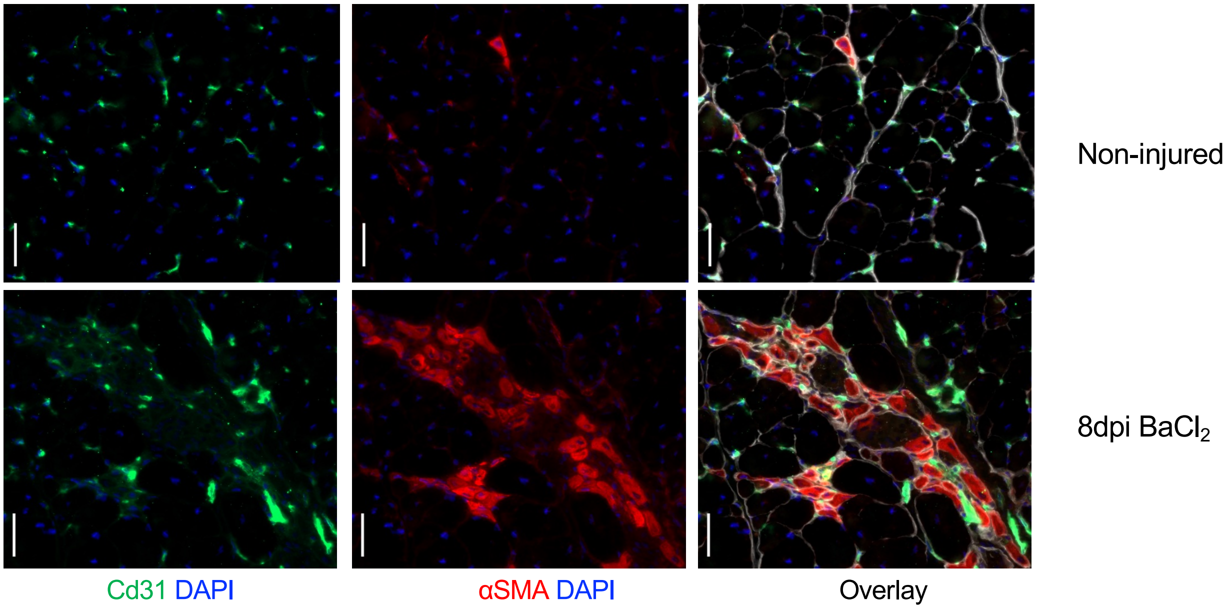

B Regenerating skeletal muscle contain an influx of macrophages

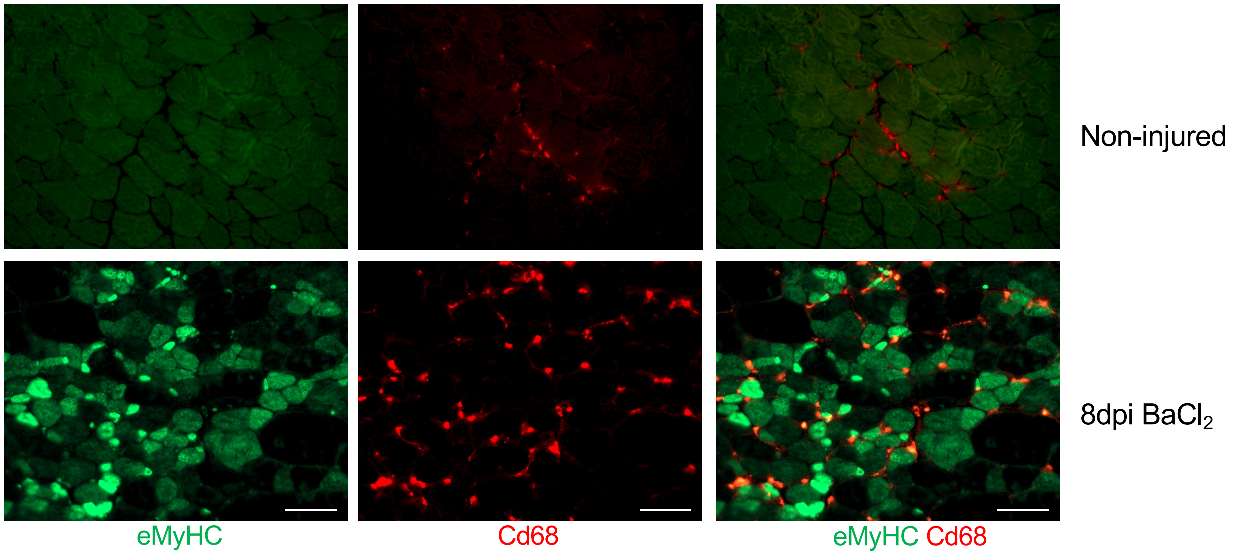

C Evan's Blue Dye is uptaken by the interstitium of regenerating skeletal muscle

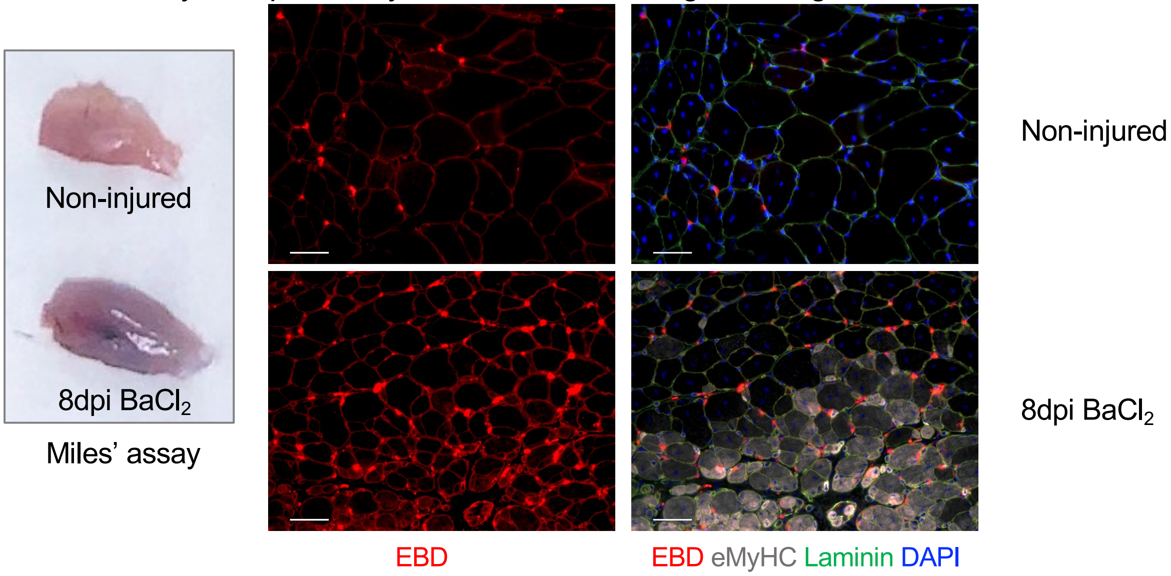

Supplement: Supplementary file 4 — Additional file 4: Figure S4. Evaluation of the regenerative microenvironment following BaCl2. A. Regenerating mdx myofibers (αSMA, red) are juxtaposed to enlarged, dilated Cd31+ capillaries (green) compared to non-regenerating muscle regions. B. Regenerating myofibers contain an influx of macrophages. eMyHC (green), Cd68 (red). Scale bars represent 20 μm. C. Images show increased Evan’s Blue Dye at the whole tissue level (blue) and within muscle cross sections (red) at 8 dpi compared to non-injured. [file 12951_2023_1994_MOESM4_ESM.pdf]
